# The Influence of Material Objects upon the Mind

**Published:** 1863-04

**Authors:** J. Alexander Davies


### Art. IV.—THE INFLUENCE OF MATERIAL OBJECTS UPON THE MIND.

By J. Alexander Davies.

The various phenomena of dreams afford peculiar illustrations of the way in which the mind is connected with its material surroundings; and this observation may be extended, and indeed with more force, to somnambulism generally. I shall not here consider these various phenomena in a physiological manner, but merely do something towards showing how the human mind is affected by man's corporeal frame.

During sleep the mind, which is never then in its reflective state, is not susceptible to external sensations. A sleeping man knows neither warmth nor cold, neither is he affected by any moderate sound, nor—and this from a physical necessity—light, inasmuch as his eyes are closed. There can be no doubt that in dreams the mind is affected by the physical frame in a manner similar to what it is by external sensations. As regards dreams—and this in common with every sleeping affection—it may be said that man never thinks, meditates, or reflects, these words having the same meaning. It is probable that this assertion will, at any rate at first sight, be considered erroneous, but I am persuaded that the experience of everyone will ultimately satisfy as to its truth. In the state of dreaming, men imagine that they are doing certain things, or merely that they are placed under certain circumstances; but no man ever dreams that he is thinking, that is, *really* thinks in the course of a dream, without immediately awaking, for if a man ever dreamt that he was thinking, he would, it is easy to perceive, *really* be thinking; and it is obvious that a *state of reflection* would immediately show him that he was in a dream, or, in other words, awake him. It will probably be thought by some that the fact (supposing it) of an individual's belief of his having reflected during dreams, is not *identical* with the fact (also supposing it) of his really *having* thought; but a minute consideration of the matter will show that the *idea* of thinking, as distinguished from a real (voluntary) act of reflection, is not a *possible* one, and, by consequence, an *impossible* condition. It is most evident that in dreams very frequently certain ideas so succeed one another as they do when reason is used; a *rational* conclusion or rational conclusions, considered *as* a conclusion or conclusions, is or are formed, and yet there never is any reason. Every rational and other conclusion is formed by *association,* and hence *involuntarily,* which process, except that the volitionary power is unemployed, tallies with the method of reasoning we use when awake, when the ideas of association are evoked by effort, the will, these, when an individual is dreaming, arising without any exertion on his part, of which, without considering them to be produced by reflection *proper*, he is nevertheless conscious. The experience of *desiring* certain things in dreams is a proof that the will is then active, whether or not it is supposed in any way to be exerted; but inasmuch as we never *perceive* the various absurdities formed by the strange mixtures of actions and things which appear to surround us, we have here another proof that the mind never assumes the reflective state, for were this the case, these absurdities could not but be discovered, and, by consequence, we could not but awake, or perceive that we were dreaming. It has been supposed by some that the mind is never in a quiescent or slumbrous condition while the body is asleep, but I apprehend that this notion cannot be received, for the reasons already brought forward to substantiate it. If it is said that men sometimes talk and walk while asleep, and moreover quote from authors and languages they are unacquainted with while awake, and all this without having any idea of it when their sleep is over, I must reply that all these phenomena occur mechanically, or when the will is for the time destroyed or rendered incapable of action; and consequently, that the non-remembrance of these things affords no analogous reason for supposing that man invariably dreams during sleep, although he seldom remembers his dreams. Whenever a man remembers having dreamt upon some subject, he also remembers that he imagined himself to possess, and a little consideration would enable him to see that he really had, the power of volition, although not that of thought or judgment. Now as the other phenomena of somnambulism occur mechanically, I apprehend that the subject of such would not upon awaking at all remember what he had said or done, inasmuch as all merely mechanical operations are only corporeal, and hence do not impress the memory, or cause the mind to exist in a certain state. It would be foolish to deny that all the phenomena of somnambulism are of a wonderful nature, and but very superficially understood; this particularly applies to cases where persons who, when awake, make use of vulgar thoughts and phrases, speak with accuracy and even refinement when asleep, and such as in the same condition quote authors and languages they do not remember when awake. A late philosopher has observed that, if gently roused when in the state between sleeping and wakefulness, we find that we are in the commencement of a dream; and also that when suddenly awakened from sleep he always found that he was in the middle of one. These facts are supposed to prove that the mind is active in sleep, and seem to be considered to hold universally; but even if this be the case, I apprehend that they do not make against the doctrine of Locke and others generally, that man never dreams without remembering his dreams, or, which is the same thing, prove that during sleep the mind is never slumbrous but always active. It is at any rate conceivable, and, from analogy to a phenomenon I shall bring forward, probable, that if these facts hold universally, they arise from the act of waking. It may be thought very unlikely that dreams would ensue in such a short space of time, but it should be remembered that men sometimes dream of experiencing, and thus really do experience, hours of exertion or pleasure, when, according to the usual standard of human sensations, they have only slept a few minutes. The phenomenon I have alluded to is found in many cases of nightmare, and consists in the agreement, at first sight very curious and paradoxical, between external sensations and the supposed realities of this species of dream, if it is peculiar to nightmare, concerning which I am at present unable to decide. I remember dreaming some years ago that I saw before me a kind of state apartment, at one end of which was something in the shape of a prison, in which I appeared to be confined. I was of course terrified, although it seemed that others were with me; and at last the heavy door was slammed—I shall never forget that slam—and the happiness beyond was shut out from my sight. After awakening, I discovered that a fire in the adjoining house had then received a sudden and desperate poke; and this fact was carelessly supposed to account for the supposed slamming of the door. There can be no doubt that the real noise was the cause of the supposed one, which, however, was in the fullest sense of the term as real to me as the mere noise of the poker had I been awake; but there is some difficulty in seeing how the supposed noise—for as regards others it may so be called, which must have come after, if not been simultaneous with, the real one—could have been produced or fitted on to the dream before I awoke, for I appeared to awake—that is, by reference to my own sensations only, no person being near me at the time—directly after or perhaps upon hearing it. It has been said that the mind during sleep is not open to external impressions and sensations: it should not, however, be supposed that these do not affect it, for the phenomenon under consideration is a satisfactory disproval of this as regards a particular class of impressions; and that the various other sensations and impressions to which man is susceptible cause the mind to assume various states during sleep, or if this notion is not liked, lead to different species of dreams, has been ascertained by experimental experience. I apprehend that the difficulty in the case in question, which may be taken to illustrate a certain class of phenomena appertaining in many cases to nightmare, disappears when narrowly inspected, and hence is more imaginary than real. A man is perhaps in the middle of a dream, and suddenly a noise is made: this immediately affects his mind, throwing a certain idea or several ideas into it, or causing it successively to exist in certain states; and then he awakes, perhaps only a few seconds after the noise was produced, or it may be as soon as the mind has become acquainted with it, which popularly may be considered simultaneously. In the dream spoken of I fancied that one door was violently slammed, and do not remember being conscious of anything besides before awakening; I might have fancied that these doors were simultaneously slammed, and then awoke without any further ideas. By analogy with the above remarks, the phenomena alluded to may, if they hold at all times and with all persons, be set down to the act of awakening; this may, as in the phenomena noticed, cause ideas successively to arise in the mind, and, as in the other cases alluded to, cause what are a few seconds to wakeful persons to appear perhaps hours.

Some effects of habit or custom which are really only physical, have been set down as extending to the mind. When an inhabitant of some country town or village first comes to London, he is much disturbed at night by the noise of the various vehicles in the streets, yet at last, as is popularly and vaguely said, gets accustomed to it. The question, how this occurs, may arise. Now I think it is evident that no explanation, except that of the growing dulness of the auditory nerves, can be found to meet it. It cannot reasonably be assumed that the curiosity excited, or any other mental state, is the cause why an individual under these circumstances is at first unable to sleep, or, at any rate, much disturbed, inasmuch as no curiosity of any kind, or any other reasoning condition of the mind, except in a very few cases, exists; the first night of a stranger's visit to London he is generally disposed to pass as serenely as possible, and hence exercises no mental exertion of any kind, not even pleasing himself with the several vagaries which imagination, that easiest of our mental efforts, would introduce. He willingly drops the faculty of attention, and hence it is worse than absurd to affirm that the sounds which he so plainly hears notwithstanding this, are intensified by curiosity or any other mental effort. After a few nights the sounds wear off, and yet his mental condition is the same as it was at first; he drops the faculty of attention, and does not find himself so disturbed; from which it follows that some physical change has been effected. It must be that his auditory nerves are not so susceptible as heretofore. When a

Londoner goes to some country part, he is surprised with what appears to him the extraordinary stillness of the place; he may know it well, and hence no particular curiosity or any other mental effort is excited, from which it is clear that the effect must be entirely owing to physical causes. His auditory nerves have been blunted by the continual din surrounding him; and hence when he enters a quiet locality, every part appears to him unusually silent, not only from comparison, but also because his auditory nerves are some time in returning to their ordinary state of susceptibility. Those who sleep within the sound of mills, waterfalls, and other noises, soon cease to be disturbed by them; and it is a general observation, that if the mill or other noise suddenly stops during the night, those who are sleeping in the vicinity immediately awake. I cannot comprehend those who would set down this phenomenon to anything mental. The individuals are asleep, and hence cannot be exerting any mental power; and besides this, they may not be dreaming, or no ideas may be passing through the mind. The cause of their awakening must be the change at once worked in their auditory nerves, in the process of their returning to the ordinary state of susceptibility: the blunting influence of a continued and monotonous sound is removed, and hence these acquire their general and intended delicacy. It is no objection to this explanation, that the way in which this operation awakens the sleeper, does not at present appear. When surrounded by company, or walking along a noisy street, it is at first found very difficult to carry on a train of thoughts, or read with any proper attention; yet a little practice enables man to engage with a tolerable amount of success in both these things. I apprehend that the explanation which has been advanced will meet these points; the auditory nreves become accustomed to or blunted by the surrounding noises, and thus the mind is not so keenly distracted as at first, although the duller sounds, which are itself existing in certain and successive states, from which it immediately returns, still do much to break the train of thought, and to prevent attentive reading. To say that curiosity, or any other mental condition, is the cause of these things, is, I think, contrary to the plainest reason. A man is in the midst of some company who are gaily talking on various matters, or he is threading his way among the crowds of London; now, perhaps, he is very desirous of carrying on a train of reflections upon some serious subject, and yet he finds that he is unable so to do. This being the case, nothing appears to me more ridiculous than to say that his attention is in either case distracted by curiosity, inasmuch as he is solely desirous of carrying on a course of reflection upon a certain subject, which may be supposed altogether unconnected with the various objects surrounding him; the same remarks apply to the attentive perusal of any work in similar circumstances; and I apprehend they prove that the cause of the disturbance experienced is entirely physical. It has been objected, that the habit of hearing the same sounds renders us susceptible to them, under certain circumstances, in an increased degree; the cases of savages and of the blind are the conditions mentioned. It appears to me that this is a most careless piece of reasoning, for it is, or appears to be, forgotten, that both savages and blind individuals put forth the faculty of attention in an especial degree; the former are particular to catch the sounds of enemies, or of animals upon which they feed, and the latter find an increase of attention to various sounds necessary to their safety. I do not know how it happens with respect to barbarous tribes, but blind persons generally, perhaps always, obtain a keener sense of hearing; perhaps the increased attention they exercise helps to effect this. Thus, were the sounds heard by savages and the blind continuous, the amount of attention they exercise would not only neutralize the blunting effect of their continuity, would not only counteract this dulness of the auditory nerves, not, however, physically, but by attending more closely to sounds really duller, but would probably render them more susceptible to them than if they were not continuous; but it should be remembered that this is not the case. Savages do not catch the sounds of enemies, or those of the footsteps of animals continuously; neither are blind persons always subjected to the bustle of a noisy street; consequently there is no similarity between the phenomena, so that the fact of these classes of persons becoming sharper to sound does not prove that continuous sound does not physically affect man by blunting his auditory nerves. The habit of hearing occasional or continuous sounds with no, or an equal, degree of attention, leads to their becoming fainter; and when certain men pay greater attention to certain sounds, either occasional or continuous, than mankind generally do, and then find that they are more susceptible to them, or hear them more plainly than others do, it should not be inferred that the mere habit they are under is the cause of their increased sensibility, inasmuch as other men under the same habit find that the same sounds become duller; consequently, it must be in the superior energy of attention that the difference lies. The savage hears the splash of the paddle, and the blind hears the rumbling of the carriage, more distinctly than those who are not concerned in these sounds; and these things simply because the attention is in both cases more energetically concerned in the inspection of the various impressions and sensations produced upon the senses, and thus propagated by means of various nerves to the soul or mind passive; or, philosophically, causing it to exist successively in certain states. A certain noise, which at first awakens from sleep, is at last found to become inefficient for this purpose; thus, those who use an alarum for the acquirement of early rising or other ends, are found to complain that they often sleep notwithstanding this precaution; that, in other words, they become accustomed to it. It may with some apparent reason here be supposed, that the effect is not physical, inasmuch as its cause is not continuous, but merely a single manifestation; but I think a close inspection of the fact proves that it cannot be owing to any mental influence. A man retires to rest without being occupied with any peculiar thoughts, and in the morning is awakened by his alarum, or in some other way; but after some time, he finds that the usual method made use of to this end fails to produce the required effect. The cause of this cannot be mental, inasmuch as every night his mind is not occupied in considering whether or not he will awake on the morrow at a certain time; and yet it is not at once clear how it can be physical. The explanation must be, that the auditory nerves are daily affected, and at first return to their usual state before the recurrence of the accustomed noise, and hence are sufficiently affected by it to awaken the sleeper; but that, after some time, they become too much blunted or deadened to assume their ordinary condition before this, and hence are not sufficiently affected by it to work the desired effect. This being the case, a different noise will frequently awaken the sleeper. As everyone must grant that during sleep the mind is not fully reflectively employed by the will, although it may be supposed that some species of reflection then sometimes takes place, it cannot be supposed that the new noise produces any reflection proper, or that extended mode of thought which an awakened individual puts forth, and hence that the mind distinguishes it, or that the sleeper awakens by a mental effort; it has already been observed, that during sleep thought or reflection never takes place. The awakenment must be a physical effect merely; the auditory nerves have been blunted by the continual recurrence of a certain noise, and hence the sleeper is not awakened; but another noise, by producing a different effect upon these nerves, works the end contemplated. And at last the latter sound is of no avail for practical purposes, as might *à priori* have been supposed. The same conditions are experienced in wakefulness; those employed in cotton mills or machine shops soon become deadened to the surrounding hum, while the visitor to such places can scarcely hear his own voice for the noise, and almost fails to perceive occasional sounds which the workmen experience in almost all their force, so accustomed have they become to what is continuously presented to their auditory nerves. I apprehend that no curiosity, or any other mental effort, can here be demonstrated to exist. Nothing is more tiresome or trying than the continual clatter emanating from boiler manufactories, and other places where the process of rivetting is often resorted to, yet the men engaged in this work soon lose their susceptibility to the particular noise, and catch each other's voices, where a stranger would hear little or nothing. This noise cannot be considered a continual hum, and because this is not the case, it is that its effect is not so complete, the auditory nerves having some time to recover in the intervals of sound. Here also no mental effort appears. It is a popular observation, that a man generally awakes at a certain time when he sets his desire upon this the previous night; and this fact may be produced as something which cannot be levelled to physical causes merely, and which bears unfavourably upon the previous observations. I see, however, no reason in this for supposing that the mind in its complete sense, that either the mental energies or the moral affections, are active during sleep: desire is a condition belonging to the latter class, and in the case in contemplation, may, I think, be supposed to produce physical effects, which result in causing awakenment at a certain hour. It is true that this case differs from those which have been noticed, in that it is based upon a certain moral condition; but I think it affords no reason for supposing, that during sleep the mind, both moral and mental, is never slumbrous, or that in the experience of dreams the faculty of reflection generally, which includes every active or real mental effort, is ever exercised. The susceptibility of the mind proper to external impressions and sensations, must be considered a passive rather than an active power, just as the force of cohesion in matter is apparently passive. It should be observed, that these terms are used merely for distinction, there being no difference in kind between active and passive power, but only in degree, condition, and, consequently, appearance. The susceptibility of the mind, equally with the cohesion of matter, is produced and maintained by some force or motion, for all force or effort is motion of some sort; and although these things differ from the process of reflection generally, or any particular branch of it, and the evident or apparent influence of gravitation where a stone is caused to fall, respectively, it cannot be made out that the terms active and passive, as applied to fall, imply anything more than human convenience. When the force of gravitation has apparently ceased, as when a ball rests upon the ground, or a book upon the table, this influence may, to suit human convenience, be termed passive, although it is evidently as active or effectual as before. I

shall only bring forward one more set of illustrations, where mere physical effects may be, and perhaps have been, considered as partly mental or moral phenomena. When a man goes out in a new garb, which perhaps may be similar to the dresses he constantly meets, he finds himself for a certain period ill at ease, although he is unconscious of any peculiar mental or moral condition; he beholds his dress, and remembers or is conscious of its newness; and these things for a little time disturb him. The fact of his consciousness does not imply any mental effort, inasmuch as this faculty, which is only continual remembrance, and hence almost instinctive, is a mental susceptibility, as is the readiness of the mind when man is awake to receive external impressions and sensations. The vision of his garb, and the consciousness of its newness, physically affect him; and his uneasiness is the greater if this differs from the dresses around him. But habit blunts these as all other sensations: a man in a smock-frock soon becomes accustomed to his dress, and would feel rather odd at first in a black coat, although these move like the rest of the world. Supposing two countrymen to be transported to London, one of whom had never seen men in black coats, if the thing were possible, and himself wearing a smock-frock, and the other to have put on a black coat for the first time, it is difficult to say whose sensations would be the most intense and strange. The influence of many new circumstances and conditions may in this way be explained.

As regards all the merely mechanical operations which man learns to perform, I am inclined to agree with those who think that all such habits are merely acquired instincts. A novice can scarcely produce any sound upon the flute, and when learning any particular tune finds it necessary to think before every note, while one accustomed or habituated to the instrument can play various pieces apparently without the exercise of any thought, and even while attentively looking at various objects, or carrying on different trains of thought. To say that in operations of this kind—I mean such as are merely mechanical—a thought, or process of thought, precedes every motion of every finger, appears to me unphilosophical, inasmuch as there is no remembrance of this, and it is difficult to understand how such should occur, and not in any wise be remembered; and besides this, it cannot be supposed that the mind thinks of two things simultaneously. It may be thought that the various extraneous reflections are carried on between the thoughts preceding the production of the several notes, but the want of consciousness respecting the latter is, I apprehend, destructive of this notion, were not the rapidity necessary to be supposed sufficient, and I am inclined to think that alone it is sufficient, to set it aside.

The doctrine of latent agencies, which do not rise into consciousness, which has been maintained by the late philosopher I have alluded to, appears to me in a high degree improbable, inasmuch as it is anything but perspicuous how the mind can in any degree or in any manner be employed without the possessor being conscious of this. To say that the mind is in some circumstances employed without this being known, seems to me as unintelligible as to affirm that a man can breathe the faintest whisper, and not be aware of the action; besides which it is absolutely impossible that any voluntary action can be performed unknowingly: the knowledge of what a person contemplates doing is necessary to the act. No man could speak or walk, or perform any other voluntary action, without knowing the moment before that he was about to do it; and similarly, no thought, which is always voluntary, can occur without it being known the previous moment that the mind was about to assume an active state. The instinctive doctrine cannot be demolished by any of these difficulties. I can see no objection in the supposition that mental efforts ultimately yield to influences of quite a different character; neither do I think it can be said that this principle, which has been branded, as it were, with the name of occult, as if that were anything against it, is unnecessary. Save as regards memory, the several members of the animal kingdom do not appear to possess any really mental faculty; every other species of knowledge seems to be derived from instinct, which term merely conveys the notion of something obscure or mystical. Instinct is a something serving as a succedaneum to mind, and appears to accomplish what mental efforts merely never would; it seems to lift man up out of the region of mind, to take him off his legs, so to speak, and thus cannot be looked upon as something contemptible and degrading. I apprehend that no series of mental efforts would ensue with a sufficiently quick succession to enable a person to play rapidly upon the violin or piano; and inasmuch as, when this is attained, no mental effort prior to every note appears upon the closest inspection, it seems to me only reasonable to suppose that the cause of the acquired faculty has been transferred to some unmental principle, which, as something manifesting itself in the several operations performed by the various animals and tribes composing the animal kingdom, is known by the name of instinct. It should not be supposed that the operations depend upon memory, for were this the case this would be discovered, which does not happen. When a person is playing upon any instrument a tune which he well knows, he is not conscious of exercising his memory even to the least degree, and hence does not exercise it; for I maintain that where a man does not perceive that he is putting forth any mental effort, there is no reason for, and every reason against, supposing that any such effort is made. Similarly, when a young person learns anything by heart, it is for the first few times necessary to exercise the memory in repeating it; but this soon gives way to mere instinct, and the rhyme or passage is rattled off without it being found requisite to obtain in any degree the assistance of this faculty.

I take it to require no sort of proof, that the mind cannot apprehend two or more impressions or sensations, or think of two things, simultaneously. When we cast our eyes around a landscape, we successively behold several objects and parts of objects; and when we select a leaf from any particular tree, it is found that we cannot view the whole of it at once, or without certain motions of the eye, and that the whole which can simultaneously become the object of our vision is the smallest spot or portion discernible; consequently, when we gaze upon objects the eye is not directed to any particular part, but a general impression is produced upon the retinas, although it is found that those parts of bodies from which light falls upon the centre of the retinas, are the most distinctly represented. Near the eye, I cannot but think that man is unable to see a surface larger than the point of a pin simultaneously, or without a motion of the eye; and even this appears too great, for when it is considered that the mind can apprehend but one impression at a time, and that it seems necessary that a point or surface should be only just perceptible to produce but one impression, the amount of surface named must be considered to require diminution, if it is allowed, as I suppose will be, that any point or surface smaller than the end of a pin is perceptible by human vision. As a general survey, I can understand that man may have the impression of several balls, for example, simultaneously; but that the mind can be actively directed to each of these objects at the same time appears to me quite as paradoxical as to affirm that man can walk two ways at once; it is impossible that the mind can simultaneously be engaged in two operations, notwithstanding it is popularly affirmed of a certain historical notability that he could both attend to what was said to him and dictate at the same time. I apprehend that it is possible barely to hear what a person is saying or reading, and to read or dictate at the same moment; but that any man could attend to what was being read or spoken, and attentively read or dictate at the same time, and thus be actively engaged in two mental operations at once, I hold to be a notion wholly absurd. It is a popular observation that a person may read merely mechanically, and have his attention directed to what another person is saying or reading, or his mind engaged in various trains of thought. Now, the process of reading certainly requires attention, which is an active state of the mind, inasmuch as the impressions of the various words upon the mind would not be produced merely by a listless gaze upon the page, although, except perhaps in very uncommon cases, where the thoughts are of a very intense kind, it would always be known that a book, with some species of character thereon, were before us; and inasmuch as the mind cannot simultaneously be active in two ways, it follows that the attention or reflection must occur between the apprehensions of the various words, and perhaps while these are being pronounced, seeing that this operation is wholly a mechanical one. Man is so accustomed to exercise some amount of attention when looking upon various objects—I mean successive attentions to various minute points upon their surfaces—that it has become almost difficult to suspend the exercise of this faculty when the experiment is necessary. Although I consider it impossible that man can attend to two or more simultaneous sensations or impressions, yet I see no reason for supposing that a plurality of such cannot at the same moment be experienced. As Locke says:* "Now, I see the white and black on this paper, I hear one singing in the next room, I feel the warmth of the fire I sit by, and I taste an apple I am eating, and all this at the same time."

It is very material to observe that this opinion was not maintained by Locke, but merely quoted by him as something with which he could not agree. He appears to me to have supposed that the various impressions and sensations produced upon the mind, as well as its several sorts of activities, are modifications of its conditions at any particular time, and thus to agree in this point with a more modern philosopher, for he remarks a little further on, "when I recollect the figure of one of the leaves of a violet, is not that a new modification of my soul, as well as when I think of its purple colour?" While I think that the fact, if such it is, of the absolutely simultaneous impression of two or more outward impressions or sensations upon the mind, cannot be demonstrated, I cannot find any reason for supposing that this notion makes against that of every passive and active condition of the mind, being only itself in various states or modifications: in the illustration advanced, and all similar, the mind may be in a combined and yet only single state, for I suppose that when closely inspected no meaning can be attached to the term indivisible, as applied either to the mental faculty

---

* *An Examination of Malebranche's Opinion*, &c., sec. 39.

or anything else with which man is acquainted. We want the power of conception to be enabled to understand this term, as well as the word unextended; and, inasmuch as this does not appear, the use of these words is of no benefit, but only produces confusion, because, in other words, no meanings or ideas are suggested to the mind by their employment. In a musical performance, where many sounds are simultaneously produced—in other words, in a concert—it is popularly supposed, not only that all the sounds reach the auditory nerves simultaneously, but also that all are impressed upon the mind at the same moment. Perhaps it is true that in the above circumstances all the sounds meet the auditory nerves at once; but this does not prove that the mind apprehends them simultaneously, inasmuch as the influence of certain sounds upon these nerves may be more active than that of others, in consequence of which the mind receives them successively. When a person hears two or more sounds successively which he does not know to have been produced at the same time and distance, he concludes that this has not been the case; but I apprehend that it is seldom considered whether different sounds do not affect the auditory nerves with a greater and lesser degree of vigour, and thus, by impressing the mind at intervals, do something towards increasing, and perhaps in some cases lessening, the spaces of time composing the phenomenon of succession. Considering it true that in a concert all the sounds simultaneously produced fall upon the auditory nerves at the same time, and moreover that these are transmitted throughout these nerves with the same velocity, and thus meet the mind, or are in a position to become mental, at once, it does not from either point follow that the mind apprehends them at precisely the same period. It may seem difficult to suppose that a great number of sounds are transmitted through the auditory nerves at the same time, and more so that these are impressed upon the mind simultaneously; but these difficulties do not suppose contradictions, and thus are not fatal, and besides this, they are the least to be put up with; for to suppose that the mind receives the various sounds of a concert successively, and that the harmony of these is perceived by reason, (for it does not appear that this could be known but by comparing the sounds in memory and that at present experienced, and that these things are not known,) is to support a doctrine of no mean difficulty, and which indeed would be fatal were it absolutely necessary to suppose that the sense of harmony is attained by a rational process, inasmuch as nothing of this sort can occur without the consciousness of the author; and, although not perhaps absolutely necessary, the probability of the sense of harmony being obtained in an unmental manner is so small, that the belief at once gives to any doctrine a very unfavourable aspect. I see no reason for supposing it impossible that the mind can compare the sounds in memory and the one in sensible experience, or that this is more than a particular state of the same, any more than I hold it absurd to suppose that a person may read or dictate, and hear, although not intelligibly or attentively, what another is reading or saying at the same time, and that this is only one condition, and as before, and in the case of a plurality of merely sensational experiences, a combined one; but it does appear to me that to suppose the mind to exist in any active or partly active state, as in the cases brought forward, without the author being conscious of this, is a notion which will not bear a candid and lively inspection. Upon the supposition that the many sounds simultaneously produced in a musical performance meet the auditory nerves at the same moment, and are transmitted through them with equal velocity, and thus meet and are apprehended by the mind at once, (for it should be observed that these two conditions are not the same, inasmuch as a number of sounds may be supposed to travel over the physical ground, or be transmitted through the auditory nerves, and thus be in a position to become mental simultaneously, and yet be apprehended by the mind successively)—it is unnecessary to conclude that any mental effort is exerted in the sense of harmony experienced. This is not discerned, and hence may be denied, yet from this it does not follow that the perception is instinctive; the succession of various sounds may thus be supposed to affect the mind in its passive state. It is common to talk of training, sometimes foolishly called educating, our various organs of sense. The eye by practice gains a facility in adjusting itself to see small objects at a considerable distance, or, to speak correctly, the owner comes to acquire an increase of power over his organs of vision by means of muscular development; similarly the organs of hearing are, or may be by practice, enabled to distinguish sounds almost similar, whether in note or degree. Thus the Indian, by putting his ear upon the ground, can tell, by the peculiar splashing of the oar, whether friends or enemies are approaching, and by the tread of the advancing or retreating animal, to what tribe it belongs; and the palate may be altered, so that food at first considered disagreeable may after some time pleasantly affect us. These phenomena are brought forward in order that an inspection of them may show that they are wholly independent of any mental influence. If some which have been noticed cannot be connected with any mental exertion, (and it is not in accordance with the philosophical method to conceive that the connexion exists, when the owner is not conscious of it,) then it may be pronounced that these phenomena are in their entirety physical.

Every person may observe that artificial scenes and written tales which are known to be fabulous, excite our sympathy as if they were real. These phenomena have been explained by the supposition that they are, for the time, believed to be realities; but I apprehend that this notion will not bear a remarkable amount of inspection, for whether an individual is torn from some fascinating book, or tapped upon the shoulder when listening to the words and beholding the actions of some artificial scene, and asked whether he believes these things to describe realities, he will at once affirm that he does not believe this, and yet may have the traces of emotion visible upon his countenance. I know it may be said that these actions restore a person to his usual condition of consciousness, and thus enable him to judge rightly, and hence that they do not prove that a man does not for the time believe in the reality of unreal tales and scenes. But this cannot be the case; for were it believed, even for a moment, that any fabulous tale or scene was real, this would have been known, and could not but have been remembered afterwards, notwithstanding the enlargement of consciousness, neither of which things occur. It appears to me that our feelings of pleasure or pain in such circumstances, are produced by the consideration that similar events to those set before us have occurred, or are now occurring. It is observable that there is nothing irrational in this consideration as such, whereas it is entirely ridiculous to suppose, even for one moment, that the fabulous things read of or witnessed are true; and thus it is improbable that this is supposed, which improbability merges into absolute negation when it is considered that the imagined belief is neither known at the time, nor remembered afterwards. It is clear, that unless the belief here adduced is experienced at these times, it must be cast aside as the other has been: I apprehend that the necessary agreement both may be and has been found. It will be considered strange that as a certain belief has been experienced, men should have forgotten this, and have supposed that they believed something else at the particular time; but it seems that this has been caused from the want of attention. When an individual is reading the account of some remarkable escape from danger, for example, which he knows to be fabulous, he is so interested with the various incidents laid before him, that he gives very little attention to the states of his mind between the apprehension of the different words, and perhaps while these are being pronounced, if the mind is then active, while the idea of the last word is fading from it; for I think that the mechanism of speech may work with the simultaneous condition of the mind in an active and passive state, although I hold it to be unphilosophical to suppose that the mind can be in more than one active state, or exert itself in more than one direction, simultaneously. If we break off abruptly from any interesting book, the contents of which have caused our feelings to be excited, we shall, if I may decide from my own impressions, be enabled to find that we have been believing similar events to have happened, or then to be taking place; and inasmuch as it is not usual to give much attention to these judgments, partly on account of the interest accruing from the pleasing volume, they are all but erased at the time, and scarcely remembered afterwards. This explanation appears to be supported by facts. In reading "Gulliver's Travels," the feelings of joy or grief are never excited by means of the several vagaries there to be found, even when these are such as would in probable cases produce them very strongly; neither if broken off in any portion of the work, are we in any degree conscious of having believed that similar events either have occurred, or are then occurring. These facts prove that the belief that similar events to those read of either have occurred or are then occurring, is necessary to the activity of the feelings. This belief cannot reasonably be called instinctive, for nothing mental is so, although many mental operations are habitual: although the quick succession of certain ideas in the mind caused by association, and the sometimes rapidity of judgment, is to all appearance mechanically produced—using this term not in its common or obvious, but in its philosophical sense—an habitual train of association or mental conclusion, must not be set down as something merely instinctive. For instinct is, as properly considered, a blind director; whereas everything mental is clear, and habit affects us only by oiling the wheels and tightening the springs of the mental machinery, which renders the mind less lumpish in some cases, or enables the operations preceding certain conclusions, and in some instances the succession of associated ideas, to proceed more rapidly, and this without rendering these conclusions or associations in any wise instinctive. When reading Poe's tale, "The Gold Beetle," the sensation of hope is strongly roused on behalf of Legrand; and many parts of the "Arabian Nights" awaken the same feeling; we know that the events described are but imaginary, nevertheless, considering it probable that similar things have occurred, or are now occurring, those feelings applicable to realities are aroused.

These remarks are capable of application to the various representations of life and character. If a person pretends to be suffering from causes which it is not probable that man ever has suffered or is then suffering from, the feelings of the audience will not be roused, neither can they be made to sympathize with apparent joys which it is not considered probable that man has ever felt, or now experiences. It is so with hope; if a man, as his part in any performance, pretends to hope for something which there is no reason for believing that man ever has hoped or is now hoping for, the real feeling is never called into activity by the audience.

When angry at being unable to produce certain results, it is sometimes the custom of man to abuse the materials which are incapable of effecting the purpose intended, or which he cannot shape or adjust in the desired manner; thus a workman angrily throws away his tools, and a student dabs his pen upon his desk, while the clerk is often found to tear in pieces the work of many days, at being unable to discover some confusion which has arisen in his calculations, and the mechanician angrily throws aside or pulls asunder the complication of wheels and springs which he cannot so combine as to effect the end in view. To say that the materials so used are for the time believed to be conscious, I take to be a notion wholly ridiculous, inasmuch as the closest inspection of the mind under these circumstances shows no signs of this belief, which could not exist without the owner possessing the consciousness of this in the same degree.

I understand not those who make any radical distinction between the various qualities of matter. By pressing upon a table or against a wall, it may be ascertained that matter possesses the quality of resistance, and by moving the hand along these objects, it is known that it also possesses that of extension. The eye informs us of colour, and the palate of taste; but because these experiences, and others like them, arise from the various sorts and motions of matter, it should not be supposed that they are different in kind to resistance and extension, to which all the primary qualities of matter may be reduced. The heat of a fire would be the same were there none to experience it; and similarly, a wall-flower would so reflect the solar rays as to produce its image upon the retina, and send forth particles of matter capable of affecting the olfactory nerves; and an apple would possess those passive forces (by which term I understand unobvious, and thus make use of it merely to suit human convenience) were there no person existing to enjoy these things. It has been said that form is an objective condition of matter and a subjective condition of the mind; and the same thing must be remarked as to extension, and with this addition, that this is also a quality of matter, which cannot be supposed of form. It cannot reasonably be maintained that colour, sound, and similar experiences, are not real qualities of matter, inasmuch as by removing the objects producing these impressions, they disappear: it is true that the motions of various sorts of matter are apprehended by man, as colour, sound, taste, and in other ways; but from this it cannot be concluded that these qualities exist only in ourselves, if any meaning is to be attached to these words. No person will, perhaps, now deny that the motions of various sorts of matter are apprehended by us in the experiences referred to; and hence it is ridiculous to maintain that colour is only something in the eye, and sound only something in the ear, if by this more is meant than that these experiences are the apprehensions, I do not say manifestations, inasmuch as this term suggests the idea of some species of change of certain motions, for every material influence *is* caused by motion. Hobbes adduces as an objection to this doctrine the fact, that people often see double, as two candles for one, and may hear one sound two or three times in echoes; but in the present state of physical science these phenomena would never be considered to prove the absolute subjectivity of the secondary qualities of matter. He says:—"It is apparent enough that the smell and taste of the same thing are not the same to every man, and therefore are not in the thing smelt or tasted, but in the men."* It is certainly probable that the motions producing these experiences are not similarly apprehended by all; but this only proves that men do not alike apprehend these motions, and not that smell and taste is in man. Because one person likes the smell or taste of something which is disagreeable to another, it does not appear necessary to suppose that they apprehend the motions differently, inasmuch as the difference of effect produced may be set down to the various characters of the human soul; and even this without supposing that these experiences are in man; and the same thing applies to the impressions of sound, heat, and others.

It may be thought that this question is merely a verbal one; but it seems to me that it is more than this, and lies in the distinction between apprehensions and manifestations: those who hold that the secondary qualities of matter are in man, appear to think that some change or manifestation takes place, whereas the doctrine supposing them to be merely apprehensions of motions renders this subjectivity unnecessary. Without the tactual power man would be unable to ascertain that resistance and extension are modes of matter; so that his knowledge of these things lies upon the same ground as does that of its secondary qualities—that is, the general basis of human experience.

The observations advanced show in some measure the con-

---

* *Human Nature,* chap. ii. sec. 9.

nexion between material objects and the mind. The want of conception in man cannot at any rate be considered favourable to the doctrine, that the mind is, as a working instrument, altogether independent of material influence; and I very much doubt whether this fact can be set down as not bearing unfavourably upon it. No just reasoner will maintain that man possesses or has possessed the faculty in point: we may take any species of imaginative writing—"Paradise Lost," "Lalla Rookh," "The Pilgrim's Progress," "Gulliver," "The Arabian Nights," "Poe's Tales," or the mystical and gloomy legends of ancient times, and find that every image or action may be, either by analysis or combination, brought into similarity with some object or phenomenon of human experience. I think that one of the best attempts at conception is to be found in Moore's "Epicurean," where is clearly seen the impossibility of man to represent scenes otherwise than by fabricating the various ideas derived from his experience. We can imagine a person to be born in space, out of view of the earth or any other material object, or to be carried to such a locality immediately after his birth. Let it now be considered whether he could think at all, in any degree, upon any object or subject. If by man we understand the human mind devoid of all material embodiment, and suppose that this could work apart from the brain, just for the sake of convenience inasmuch as this is not probable, it does not follow that it would even have the consciousness of its own power, because no exertion could be supposed, and the consciousness of future power is only the remembrance of past exertion, and the idea that the ability for this will continue: no object would be presented to the mind, and consequently there would be no subject for it to think upon. It is here of course supposed that the faculty of volition is present with the mind, and, which is probably possible, that it can exist in space. In understanding by man the mind connected with the animal frame, and thus supposing impossibilities for the sake of the illustration, it is obvious that the human body would afford much matter for reflection; and also that many points of the phenomena of the human mind could in this way be studied. It is not clear to what extent the knowledge of the human frame would enable a man to obtain that of other material objects, but it may, I think, be presumed that without this a man would be unable to think at all. He would possess neither objective or subjective matter for reflexion; and were this the case, the impress of matter would perhaps be necessary to render his mind proximately powerful, even as regards his consciousness of this, for the remembrance and expectation described could not occur but when the mind was in its proximate state.

